# Polymorphism rs259983 of the *ZNF831* gene is associated with the risk of anemia in pregnant women with gestational diabetes

**DOI:** 10.1186/s43042-025-00723-6

**Published:** 2025-06-03

**Authors:** Nataliia Karpova, Olga Dmitrenko, Ekaterina Arshinova, Malik Nurbekov

**Affiliations:** Federal State Budgetary Scientific Institution, Research Institute of General Pathology and Pathophysiology, Moscow, Russia

**Keywords:** Anemia, Gestational diabetes mellitus, Zinc-binding proteins, *ZNF831*, Rs259983, Polymorphism

## Abstract

**Background:**

Iron deficiency is a cause of anemia in pregnant women. Iron metabolism is closely related to zinc levels and the state of zinc-containing proteins. Zinc-binding proteins (*ZNFs*) can also bind other metal ions. Genetic factors can also be a factor leading to unstable iron levels in the blood.

**Results:**

The aim of this study was to investigate the relationship between rs259983 of the zinc finger protein 831 (*ZNF831*) gene and anemia in pregnant women with gestational diabetes mellitus (GDM). The PCR test system with Taq-Man samples was developed for genotyping rs259983 of the *ZNF831* gene. As a result, an association was found between rs259983 of the *ZNF831* gene and the risk of anemia. According to in silico analysis, the *ZNF831* protein is able to bind iron (FE, 0.69) and other ions, which may play an essential role in anemia pathogenesis.

**Conclusions:**

Carriers of the C allele in the homozygous state rs259983 of the *ZNF831* are at greater risk of developing anemia. Further studies are required to assess the effect of *ZNF831* polymorphisms on the risk of pregnancy pathologies.

## Introduction

Worldwide 36.5% of pregnant women and 29.6% of women aged 15–49 are estimated to be anemic [1]. The Ministry of Health of the Russian Federation reports that over the past ten years, the incidence of anemia has increased 6.3 times, and the average level of anemia in 2017 is 32% [2]. Anemia in chronic diseases resistant to iron therapy is more expected to develop in pregnant women with carbohydrate metabolism disorders (such as gestational diabetes mellitus, GDM) due to glucose toxicity and lipotoxicity [3]. Iron deficiency is the cause of anemia in pregnant women in about 90% of cases [2]. The leading sign of iron deficiency anemia (IDA) in pregnant women is a decrease in hemoglobin (HB) < 110 g/l, which may be due to reduced iron intake or increased iron loss, iron malabsorption, chronic blood loss, pregnancy, or infections and inflammatory diseases [4].

To date, iron metabolism has been most thoroughly studied and the proteins involved in iron absorption, transport, and excretion have been identified [5]. Genetic risk factors can also lead to impaired iron metabolism in the blood, provoking the development of IDA. Studies have shown that the variability of iron concentrations is partially genetically determined and the heritability of the trait is estimated at 20–30% [6]. A meta-analysis of genome-wide association study (GWAS) by Bell et al. (2021) revealed 56 loci containing genes responsible for iron homeostasis, including rs3747602 *ZNF500* and rs469882 *ZNF644* [7].

Iron metabolism is closely related to zinc levels and the state of zinc-containing proteins and enzymes. Zinc deficiency can affect the development of anemia [8]. Literature data suggest that serum zinc levels correlate with markers of iron homeostasis [9–11]. Previous studies have shown that 4–10% of genes in the body encode zinc-binding proteins (*ZNFs*), which belong to the largest family of transcription factors and play an important role in protein catalytic activity, stability, and folding [12, 13].

The *ZNF831* protein is one of them and is encoded by the *ZNF831* gene, which is located on chromosome 20q13.32. Few studies have shown that the rs259983 *ZNF831* variant has a significant association with preeclampsia, high blood pressure, and a high risk of metabolic diseases [14–18]. There is no scientific data on the association of rs259983 and other *ZNF831* gene polymorphisms with anemia. Since it was shown that many previously annotated zinc-binding proteins can also bind other metal ions [19, 20], we suggested that *ZNF831* can bind iron ions, whose deficiency plays an important role in the pathogenesis of IDA. Therefore, the aim of this study was to investigate the relationship between rs259983 of the *ZNF831* gene and IDA in pregnant women with GDM.

## Methods

The study used 423 samples of DNA extracted from the venous blood of pregnant women with GDM of indeterminate ethnicity (due to the ethical standards of the local medical register), which were regularly observed during the ongoing pregnancy and delivered in 2019–2022 in the maternity ward of the Bauman State Clinical Hospital No. 29 of the Moscow City Health Department. The age of the study participants was comparable. The Ethics Committee of the Perinatal Center of the Bauman Moscow Clinical Hospital No. 29 approved the study protocol. The Ethics Committee of the Research Institute of General Pathology and Pathophysiology approved the study protocol and each participant gave written informed consent in accordance with the Helsinki Declaration.

The diagnosis of GDM was established on the results of the OGTT (oral Glucose tolerance test) in accordance with the recommendations of the Association of Groups for the Study of Diabetes in Pregnant Women (IADPSG) and the criteria of the Russian National Consensus, clinical recommendations “Gestational diabetes mellitus: diagnosis, treatment, postpartum observation” [21, 22].

The anemia diagnosis was defined using clinical guidelines “Iron deficiency anemia” [23].

Pregnant women with cancer of any localization, autoimmune and inflammatory diseases were excluded from the study.

The samples were divided into two groups: a group of pregnant women with IDA (*n* = 249) and a group of pregnant women without IDA (*n* = 174).

### Collection, transportation and storage of samples

Biological blood samples were obtained by taking blood from the ulnar vein just before delivery. Blood in a 5 ml volume was immersed in sterile tubes with 50 µl 0.5 M EDTA, pH = 8.0, which was used as an anticoagulant. After collection, the samples were stored at + 4 °C.C and transported to the laboratory at the same temperature. After entering the laboratory for the regulation of reparative processes, genomic DNA (gDNA) from venous blood samples was isolated by Maniatis phenol chloroform extraction, followed by precipitation of gDNA with ethanol and elution with TE buffer (Eurogen). Qualitative and quantitative assessments of the isolated gDNA were performed on a NanoDrop 1000 spectrophotometer in the dsDNA-50 (double-stranded DNA) analysis mode. The analysis was conducted in the NanoDrop 1000 operating software version 3.8.1. gDNA was stored at − 20 °C before genotyping.

### PCR conditions with Taq-man samples

Primers and TaqMan probes were previously selected [18] and synthesized by Evrogen, Russia. The sequences are presented in Table 1.

**Table 1:** Sequences of primers and TaqMan samples for genotyping rs259983 of the *ZNF831* gene

Primer type	Sequence (5’ → 3’)
Forward	GAGGAAGGATGTGGCGAGG
Reverse	GAAGCTGTGGTCAGGAGGAG
TaqMan probe for the A allele	FAM-CTTGTCTCATGG**A**CGCTCTTGATCG-BHQ1
TaqMan probe for the C allele	HEX-CTTGTCTCATGG**C**CGCTCTTGATCG-BHQ1

The alleles that distinguish TaqMan Probes are indicated by bold, underline

Amplification was performed in a CFX 96 Touch programmable real-time thermal cycler (Bio-Rad, USA). The real-time polymerase chain reaction (RT-PCR) reaction mixture for one 25 µl sample contained 20 ng gDNA, 1X qPCRmix-HS (Evrogen, Russia), 200 nM of direct primer, 200 nM of reverse primer, and 250 nM of each TaqMan sample.

The PCR conditions were as follows: initial denaturation for 5 min at 95 °C to activate Taq polymerase; then 40 cycles, which included: 30 s at 95 °C, 30 s at 60 °C, and 30 s at 72 °C, followed by fluorescence measurement. The data obtained were analyzed using CFX Manager TM (Bio-Rad) software. To eliminate possible errors in genotyping, we retested 30% of randomly selected samples. The results of repeated analysis showed that there were no genotyping errors.

### Bioinformatic analysis

Metal ion binding by the *ZNF831* protein was predicted using the MeBiPred program [24]. Access to the MeBiPred server was obtained using services.bromberglab.org/mebipred.

Possible binding sites of Fe2 + and Fe3 + ions by *ZNF831* protein were predicted using the MIB2 program [25].

### Statistical analysis

Statistical analysis was performed using SPSS 17.0 (SPSS, Chicago, IL, USA) and R 4.0.4 (R Foundation for Statistical Computing, Vienna, Austria). Differences in the frequencies of alleles and genotypes between the IDA + and IDA− groups were analyzed using the Hardy–Weinberg equilibrium test using the Pearson chi-square test separately in the IDA + and IDA− groups in the SNPassoc R package (R 4.0.4). It was assumed that the association could be calculated if there were no significant deviations from the Hardy–Weinberg equilibrium in the control group of IDA− (*p* > 0.05). Deviations from the Hardy–Weinberg equilibrium in the IDA + group may indicate an association of the analyzed polymorphism with IDA.

## Results

**Fig. 1:**
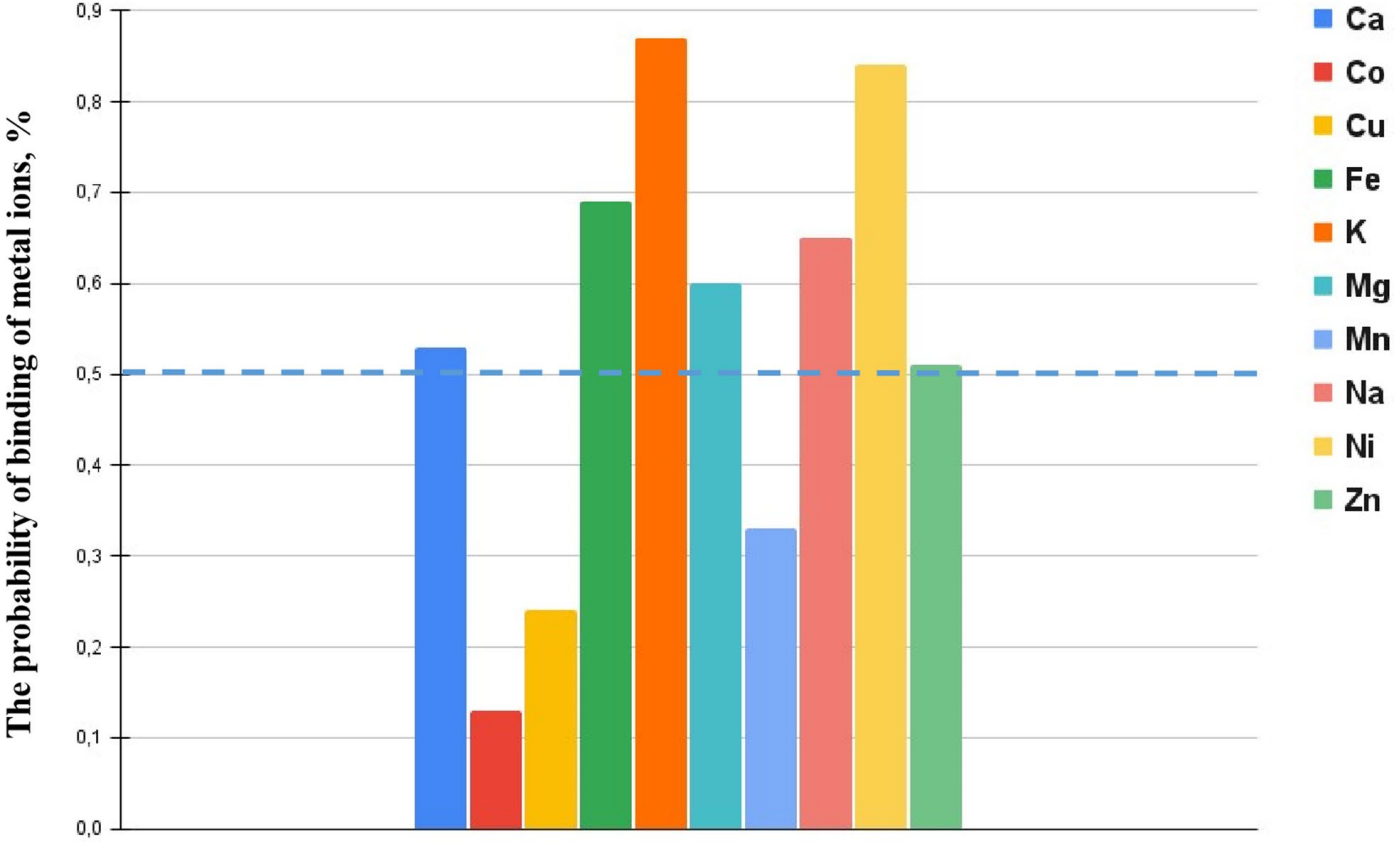
Prediction of the ability of *ZNF831* protein to bind metal ions. The threshold level is marked with a blue dashed line = 0.50

The study of *ZNF831*’s ability to bind metal ions, including zinc, may be related to anemia, since metal-binding proteins play a key role in the homeostasis of metals, such as iron and zinc, which are essential for hematopoiesis. Impaired function of *ZNF831* can affect the metabolism of iron, a key component of hemoglobin, or zinc-dependent cell division and differentiation processes, which can ultimately contribute to the development of anemia. Analysis performed using the MeBiPred program showed that the *ZNF831* protein is able to bind not only zinc (ZN, 0.51), but also calcium (Ca, 0.53), magnesium (Mg, 0.6), sodium (Na, 0.65), iron (FE, 0.69), nickel (0.84), and potassium (K, 0.87) (Fig. 1).

**Fig. 2:**
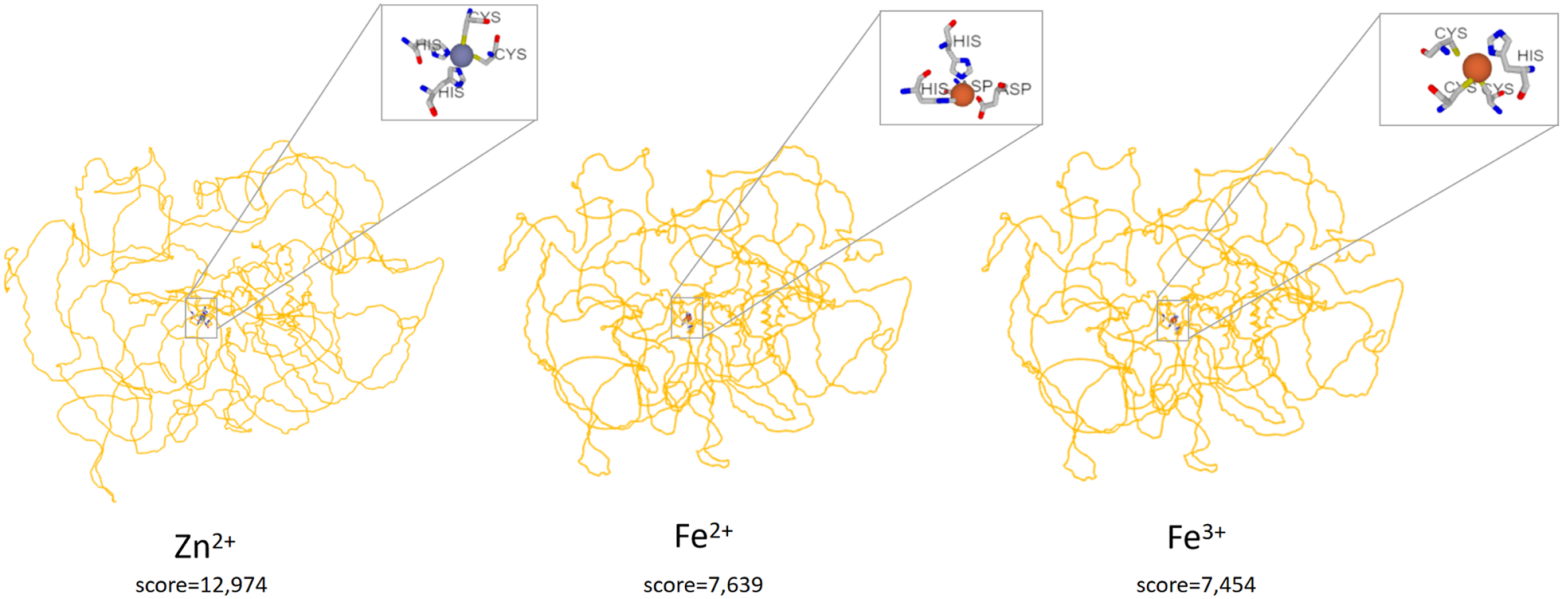
A possible binding site for Zn2 +, Fe2 +, and Fe3 + ions by the *ZNF831* protein. Alpha helices are marked with yellow helical lines; unstructured areas are marked with yellow lines without pronounced structural elements

Using the MIB2 program, we were able to predict the binding site of metal ions Zn2 +, Fe2 +, and Fe3 + by the *ZNF831* protein. Suggested metal ion binding sites are located in the center of the *ZNF831* protein near the “zinc fingers” (Fig. 2).

Genotyping of samples from 249 patients with a combination of GDM and IDA and 174 pregnant women with GDM without IDA was performed.

Analysis of the frequency of rs259983 alleles and genotypes of the *ZNF831* gene revealed a deviation from the Hardy–Weinberg equilibrium (Table 2). Differences were found in the frequency of alleles and genotype distribution between the IDA + and IDA− groups. The occurrence of the homozygous CC genotype and the C and rs259983 allele in the IDA + group of pregnant women was higher than in the IDA− group (7.2% and 0%; 20% and 16.38%, respectively, *p* < 0.005). The absence of homozygotes for the CC genotype may be the reason for deviation from the Hardy–Weinberg equilibrium in the IDA− group.

**Table 2:** Genotype distribution, allele frequency, and *p* value of the Hardy–Weinberg equilibrium for the IDA + and IDA− groups

Group	Distribution of genotypes, *n* (%)	Allele frequency	*p* value^1^			
	AA	AC	CC	A	C	
IDA +, *n* = 249	168 (68)	63 (25)	18 (7)	80	20	**0.002**
IDA−, *n* = 174	117 (67)	57 (33)	0 (0)	84	16	**0.005**

^1^*p* value values > 0.05 are marked in bold

Association analysis revealed the association of *ZNF831* rs259983 polymorphism with IDA in women with GDM in codominant, recessive, and log-additive models (Table 3). The recessive model best describes the data obtained, since it has the lowest AIC index (557.4). However, it was not possible to calculate the odds ratio (OR) for this model due to the absence of the CC genotype in the IDA− group. According to the log-additive model, carriers of the rs259983 allele C of the *ZNF831* gene have a 1.25-fold increased risk of IDA (OR = 1.25, 95% CI = 0.88–1.78 *p* = 0.0001).

**Table 3:** Association of rs259983 *ZNF831* gene polymorphism with IDA in pregnant women with GDM

Inheritance model	Genotypes	IDA +, *n* = 249	IDA−, *n* = 174	OR (95%CI)^1^	*p* value^2^	AIC^3^
Codominant	AA	168 (68)	117 (67)	1.00	**0.0001**	558.0
	AC	63 (25)	57 (33)	0.77 (0.50–1.18)		
	CC	18 (7)	0 (0)	–^4^		
Dominant	AA	168 (68)	117 (68)	1.00	0.961	577.0
	AC + CC	81 (33)	57 (33)	0.99 (0.65–1.50)		
Recessive	AA + AC	231 (93)	174 (100,0)	1.00	**0.00008**	**557.4**
	CC	18 (7)	0 (0)	–^4^		
Overdominant	AA + CC	186 (75)	117 (68)	1.00	0.095	574.3
	AC	63 (25)	57 (33)	0.70 (0.45–1.06)		
Log-additive	0, 1, 2	249 (59)	174 (41)	1.25 (0.88–1.78)	**0.0001**	575.4

^1^The odds ratio (OR) cannot be calculated if one of the values is 0;

^2^*p* value values > 0.05 are marked in bold;

^3^The AIC model with the lowest AIC value was considered the best among statistically significant models and is highlighted in bold;

^4^The value cannot be calculated because one of the values is 0

In our previous study, we identified an association of rs259983 with the risk of developing superimposed preeclampsia [18]. We suggested that rs259983 affects the risk of developing not only superimposed preeclampsia, but also hypertension in general. Therefore, to identify the risk of anemia in pregnant women with GDM, we performed additional association calculations. For this purpose, we selected only women without arterial hypertension (AH) (*n* = 345) and divided them into subgroups IDA + (*n* = 183) and IDA− (*n* = 162). For new subgroups, the distribution of genotypes and alleles was estimated to correspond to the Hardy–Weinberg equilibrium (Table 4). As in the previous evaluation, the Hardy–Weinberg equilibrium was not observed in the control group (*p* = 0.008), which we attribute to the absence of the CC genotype in the IDA− subgroup.

**Table 4:** Genotype distribution, allele frequency, and *p* value of the Hardy–Weinberg equilibrium for IDA + and IDA—groups in pregnant women without hypertension

Group	Distribution of genotypes, *n* (%)			Allele frequency, %		*p* value^1^
	AA	AC	CC	A	C	
IDA +, *n* = 183	129 (70)	45 (25)	9 (5)	83	17	0.07
IDA−, *n* = 162	108 (67)	54 (33)	0 (0)	83	17	**0.008**

^1^*p* value values > 0.05 are marked in bold

Association analysis (Table 5) revealed a statistically significant relationship between rs259983 and anemia in pregnant women with GDM without hypertension in the following inheritance models: codominant (*p* = 0.003; AIC = 469.2), recessive (*p* = 0.004; AIC = 469.4) log-additive (*p* = 0.003; AIC = 481). The codominant model is the best based on the AIC (469.2) indicators, since the OR cannot be calculated if one of the values is 0, it was obtained only for the log-additive model: 1.04, 95% CI 0.70–1.56). Thus, the presence of the C allele increases the risk of anemia by 1.04 times in pregnant women with GDM without hypertension.

**Table 5:** Association of rs259983 *ZNF831* gene polymorphism with IDA in pregnant women with GDM without hypertension

Inheritance model	Genotypes	IDA +, *n* = 183	IDA−, *n* = 162	OR (95%ДИ)^1^	*p* value^2^	AIC^3^
Codominant	AA	129 (71)	108 (67)	1.00	**0.003**	**469.2**
	AC	45 (25)	54 (33)	0.7 (0.44–1.12)		
	CC	9 (5)	0	–^4^		
Dominant	AA	129 (70)	108 (67)	1.00	0.44	480.4
	AC + CC	54 (30)	54 (33)	0.84 (0.53–1.32)		
Recessive	AA + AC	174 (95)	162 (100)	1.00	**0.004**	**469.4**
	CC	9 (5)	0 (0)	–^4^		
Overdominant	AA + CC	138 (75)	108 (67)	1.00	0.007	477.8
	AC	45 (25)	54 (33)	0.65 (0.41–1.04)		
Log-additive	0, 1, 2	183 (53)	162 (47)	1.04 (0.7–1.56)	**0.003**	**481.0**

^1^The odds ratio (OR) cannot be calculated if one of the values is 0;

^2^*p* value values > 0.05 are marked in bold;

^3^The AIC model with the lowest AIC value was considered the best among statistically significant models and is highlighted in bold;

^4^The value cannot be calculated because one of the values is 0

## Discussion

To better understand the contribution of molecular genetic risk factors to the development of anemia, we studied the relationship between rs259983 of the ZNF831 gene and this pathology in pregnant women with GDM. The results of our study indicate that carriers of the C allele in the rs259983 homozygous state of the ZNF831 gene are at a higher risk of developing anemia. The probability of developing IDA increased in carriers of the allele C by 1.25 times.

During pregnancy, in many cases IDA occurs due to the increasing iron demand in the formation of the fetoplacental complex and the redistribution of nutrient intake in favor of the fetus [1, 26, 27]. A decrease in the concentration of iron in the blood serum, bone marrow, and depot (liver, spleen, etc.) leads to a violation of hemoglobin synthesis [28]. An essential component of heme is iron (Fe^2+^). When it is deficient, heme synthesis is slow, and its amount is insufficient to perform physiological functions [29].

The ability of *ZNF831* to bind metal ions predicted by MeBiPred is confirmed by an annotation in the “Gene Ontology” (GO) database (GO:0046872) [30].

A significant part of the body’s proteome consists of metal-binding proteins that have the ability to bind to at least one metal ion and play a critical role in various functions, including catalytic and structural roles [31].

Zinc-binding proteins mainly interact with zinc ions, which play a crucial role in their structure and function. These proteins often contain two cysteines and two histidines, which coordinate a single zinc ion, forming a stable structure necessary for binding to nucleic acids [32]. The main ion that ensures their structural integrity and functionality is zinc. However, in some cases, they can also bind to other metals, such as iron, nickel, mercury, and cadmium. Replacing zinc ions with other metals can affect the activity of these proteins and be used as a method of inhibiting their functions. Replacing Zn^2+^ with Ni^2+^ or Hg^2+^ can change the conformation of protein and its interactions with nucleic acids [33, 34]. Replacement of zinc with iron in zinc-binding proteins can lead to a change in the protein conformation and a decrease in its ability to bind to nucleic acids. In addition, interaction with iron can cause oxidative stress. In turn, oxidative stress negatively affects ZNF activity and can lead to disturbances in cellular processes, such as transcription and DNA replication [35]. It can be assumed that the region near the alpha helices of *ZNF831* is the active center and forms the structure of “zinc fingers”. This structure is typical for transcription factors (TF). The GO database indicates that *ZNF831* is indeed TF and binds to nucleic acids. Chromatin Immunoprecipitation (ChIP) and luciferase reporter assays have demonstrated that *ZNF831* can directly bind to one specific region of the STAT3 promoter and induce inhibition of STAT3 transcription [36]. Thus, zinc finger proteins can not only bind to metal ions, but also actively influence the expression of many genes, including those that support normal iron homeostasis.

Genetic variants of genes can lead to various changes, including changes in the three-dimensional structure of the protein and the active center, affect affinity for metal ions, gene expression, alternative splicing, etc. [37–40]. The rs259983 polymorphism is located in the 3rd of 8 exons of the *ZNF831* gene and the beginning of the regulatory element EH38E3444973 (chr20: 59,160,309–59,160,638), which has the signature of an enhancer independent of cell type [41]. *ZNF831* has several isoforms, and the rs259983 polymorphism is located in the exon region of the transcript ENST00000637017. 1 and the 5 ‘ untranslated regulatory region of the transcript ENST00000371030.4. Data on the expression of individual transcripts are not available in databases. It is possible that rs259983 affects protein isoforms in different ways: in the NP_001371283.1 isoform, it affects amino acid substitution, and the expression of the NP_848552.1 isoform. According to the “ Variant Effect Predictor “ in the ENSEMBL database, the shorter transcript ENST00000371030.4 is canonical, and most likely the rs259983 polymorphism affects the expression and protein levels of the *ZNF831* gene [42, 43].

Prior to this study, no data existed on the relationship between rs259983 of the ZNF831 gene and IDA in pregnant women with GDM. We analyzed the samples from recruited participants, who were evaluated and followed up in the same health care facility through the whole pregnancy period. In the result of this research the Taq-Man PCR system for genotyping rs259983 of the *ZNF831* was developed and implemented. The main limitations of this study are the small sample size of the study groups. Also, only patients with gestational diabetes mellitus were included in the study. Another limitation of the study was the lack of data on such parameters as ferritin, hematocrit, red blood cell count, and changes in hemoglobin levels during pregnancy.

## Conclusions

Our study found an association between rs259983 of the *ZNF831* gene and the risk of anemia. Carriers of the C allele in the rs259983 homozygous state of the *ZNF831* gene are at greater risk of developing anemia. Despite the limitations that may have affected the results of the study, the data obtained indicate the need for further study of the association of the rs259983 polymorphism of the *ZNF831* gene with IDA during pregnancy with a parallel study of other genetic variants of the *ZNF831* gene that may also affect the development of this pathology. This will make it possible to better assess the contribution of *ZNF831* polymorphic variants to the pathogenesis of IDA in the Caucasian population of the Russian Federation.

## Data Availability

The datasets used or analyzed during the current study are available from the corresponding author upon reasonable request.

## Abbreviations

dsDNA: Double-stranded DNA
GDM: Gestational diabetes mellitus
gDNA: Genomic DNA
GO: Gene ontology
GWAS: Genome-wide association study
HB: Hemoglobin
IDA: Iron deficiency anemia
IADPSG: Association of Groups for the Study of Diabetes in Pregnant Women
OGTT: Oral glucose tolerance test
OR: Odds ratio
RT-PCR: Real-time polymerase chain reaction
TF: Transcription factors
*ZNF831*: Zinc finger protein 831
*ZNFs*: Zinc-binding proteins
